# Targeted next-generation sequencing of 565 neuro-oncology patients at UCLA: A single-institution experience

**DOI:** 10.1093/noajnl/vdaa009

**Published:** 2020-01-29

**Authors:** Matthew S Ji, Blaine S C Eldred, Regina Liu, Sean T Pianka, Donna Molaie, Bryan Kevan, Stephanie Pan, Thomas J Lai, Nhung T Nguyen, Frances E Chow, William H Yong, Christopher D Cox, Devin N Reeh, Tie Li, Linda M Liau, Phioanh L Nghiemphu, Timothy F Cloughesy, Gang Li, Albert Lai

**Affiliations:** 1 UCLA Department of Neurology, Los Angeles, California; 2 UCLA Department of Neurosurgery, Los Angeles, California; 3 UCLA Department of Biostatistics, Los Angeles, California; 4 UCLA Department of Mathematics, Los Angeles, California

**Keywords:** CNS tumors, genomic profiling, glioma, glioblastoma, targeted next-generation sequencing

## Abstract

**Background:**

Targeted next-generation sequencing (NGS) is frequently obtained at the University of California, Los Angeles (UCLA) for clinical characterization of CNS tumors. In this study, we describe the diagnostic reliability of the Foundation Medicine (FM) targeted NGS platform and its ability to explore and identify tumor characteristics of prognostic significance in gliomas.

**Methods:**

Neuro-oncology patients seen at UCLA who have received FM testing between August 2012 and March 2019 were included in this study, and all mutations from FM test reports were recorded. Initial tumor diagnoses and diagnostic markers found via standard clinical methods were obtained from pathology reports. With overall and progression-free survival data, elastic net regularized Cox regression and Cox proportional hazards models were used to determine whether any mutations of unknown significance detected by FM could predict patient outcome in glioblastoma (GBM).

**Results:**

Six hundred and three samples tested by FM from 565 distinct patients were identified. Concordance of diagnostic markers was high between standard clinical testing methods and FM. Oligodendroglial markers detected via FM were highly correlated with 1p19q codeletion in *IDH* mutated gliomas. FM testing of multiple tumor samples from the same patient demonstrated temporal and spatial mutational heterogeneity. Mutations in *BCORL1*, *ERBB4*, and *PALB2*, which are mutations of unknown significance in GBM, were shown to be statistically significant in predicting patient outcome.

**Conclusions:**

In our large cohort, we found that targeted NGS can both reliably and efficiently detect important diagnostic markers in CNS tumors.

**Key Points**

1. Targeted next-generation sequencing (NGS) can reliably and efficiently detect important diagnostic markers in CNS tumors.
2. Mutations detected by targeted NGS can be used as surrogates for 1p19q codeletion testing.
3. Targeted NGS of multiple tumor samples from the same patient can be used to assess temporal and spatial mutational heterogeneity.
4. Targeted NGS of CNS tumors can be used to detect mutations that may represent potential prognostic markers and therapeutic targets.

**Importance of the Study**

Molecular characteristics of CNS tumors have been increasingly integrated in neuropathological diagnosis. Standard clinical methods, such as immunohistochemistry and targeted sequencing, require increased consumption of valuable patient tumor samples and can be more time consuming in aggregate. Targeted next-generation sequencing (NGS) can detect many of these diagnostic markers simultaneously from a single tumor sample, which saves tumor sample and time. This retrospective, descriptive study represents one of the largest studies to describe the utility of targeted NGS in the context of clinical neuro-oncology.

According to 2017 estimates, approximately 24,000 individuals living in the United States or 1.4% of all newly diagnosed cancer patients will be diagnosed with a primary malignancy of the CNS.^1,2^ In adults, gliomas account for approximately 75–81% of all primary CNS malignancies, with the majority being classified as diffuse gliomas.^2–4^ In 2016, the WHO implemented major revisions to the diagnostic criteria for tumors of the CNS.^5,6^ Most notably, diffuse gliomas need to be separated into *isocitrate dehydrogenase* (*IDH*) wild-type and *IDH* mutant gliomas, and *IDH* mutant gliomas need to be further separated into astrocytomas and oligodendrogliomas based on 1p19q codeletion status.

As the Foundation Medicine (FM) targeted next-generation sequencing (NGS) platform has become part of the routine molecular workup of diffuse gliomas at the University of California, Los Angeles (UCLA), we wanted to conduct a descriptive examination of this tool within UCLA’s patient cohort.^7,8^ The first objective was to assess the concordance between the results reported by standard clinical methods, such as immunohistochemistry (IHC), fluorescent in situ hybridization (FISH), and PCR, and by FM to confirm the reliability of the FM targeted NGS platform. The second objective was to assess the ability of the mutations reported by FM to enhance the molecular diagnosis for oligodendrogliomas. According to the WHO 2016 updated CNS tumor classification criteria, an oligodendroglioma diagnosis is made when there is a 1p19q codeletion in the setting of *IDH* mutations.^5,6^ Currently, FM does not test for 1p19q codeletion but does test for mutations commonly associated with oligodendrogliomas, such as the *hTERT* promoter mutation, which could serve as a surrogate for 1p19q codeletion.^9–11^ The third objective was to describe intrapatient tumor mutational heterogeneity (ie, spatial differences and treatment associated temporal changes in the tumor mutational landscape) by comparing multiple samples tested by FM from the same patient.^12,13^ The last objective was to identify mutations detected by FM that are currently of unknown significance in the context of glioblastoma (GBM) and may potentially predict patient outcome and serve as future therapeutic targets.

## Methods

### Patient Cohort and Standard Neuropathological Testing

Consecutive UCLA neuro-oncology patients who received FM genomic profiling testing between August 2012 and March 2019 and had available test reports in the FM online patient database were included in this study. All patients provided informed consent under a UCLA Institutional Review Board-approved protocol. FM utilizes a hybrid-capture, NGS method.^7^ Patients included in this study received one of the following genomic profiling assays: FoundationOne, FoundationOne Cdx, FoundationOneHeme, and FoundationACT (Supplementary Methods).^7,14,15^ Patient samples tested by FM were designated as pretreatment samples if they were obtained from patients before being exposed to any treatments such as radiation and chemotherapy, or post-treatment samples if they were obtained after treatment exposure. All mutations found in each sample’s test report, including those of unknown significance, were recorded.

Initial diagnoses of patients determined by histopathological evaluation and molecular testing of surgically biopsied or resected tumor were recorded from pathology reports found in the UCLA online patient database. For patients diagnosed prior to 2016, glioma diagnoses were not adjusted to conform to the 2016 WHO guidelines for CNS tumors.^5,6^ Molecular data, when available, included *MGMT* methylation via methylation specific real-time PCR (RT-PCR), 1p19q codeletion via FISH, R132H mutations in *IDH1* via IHC, any codon 132 mutation in *IDH1* via PCR, any codon 172 mutation in *IDH2* via PCR, *TP53* mutations via IHC, *ATRX* mutations via IHC, *EFGR* amplification via FISH, and EGFR variant III (EGFRvIII) via RT-PCR and IHC. 1p19q codeletion status was interpreted by differently applied thresholds depending on the institution where testing was performed. At UCLA, at least 50% of tested cells needed to show both deletion of 1p and 19q, while this threshold was as low as 30% at other institutions. The exact percentages of cells harboring the 1p and 19q deletions were often not described in pathology reports but were recorded when available.

### Statistical Analysis of Mutations of Unknown Significance in Predicting Patient Outcome in GBM

An elastic net regularized Cox regression model was used to select for mutations of unknown significance detected by FM that were important to overall survival (OS) and progression-free survival (PFS), independent of other clinical covariates.^16^ Only primary *IDH* wild-type GBM patients who received upfront temozolomide and radiation treatment before first tumor progression were included in this analysis. OS was defined as the time between the date of initial diagnosis and the date of death. PFS was defined as the time between the date of initial diagnosis and the date of first tumor progression. The response assessment in neuro-oncology (RANO) criteria was used by clinicians to determine tumor progression. PFS was censored for patients who did not have RANO criteria confirmed progression events. OS was censored for those without a confirmed date of death before September 1, 2019. Mutations selected by the elastic net model were studied together with important clinical covariates already associated with patient outcome using a Cox proportional hazards multivariate model. These covariates included sex, age, extent of initial tumor resection (EOR), KPS, and *MGMT* methylation. The prognostic power of selected mutations for predicting OS and PFS were further assessed using an R-squared statistic for the Cox model and Kaplan–Meier curves.^17,18^*p* Values less than .05 were considered statistically significant.

## Results

### Cohort Characteristics

**Figure 1.**
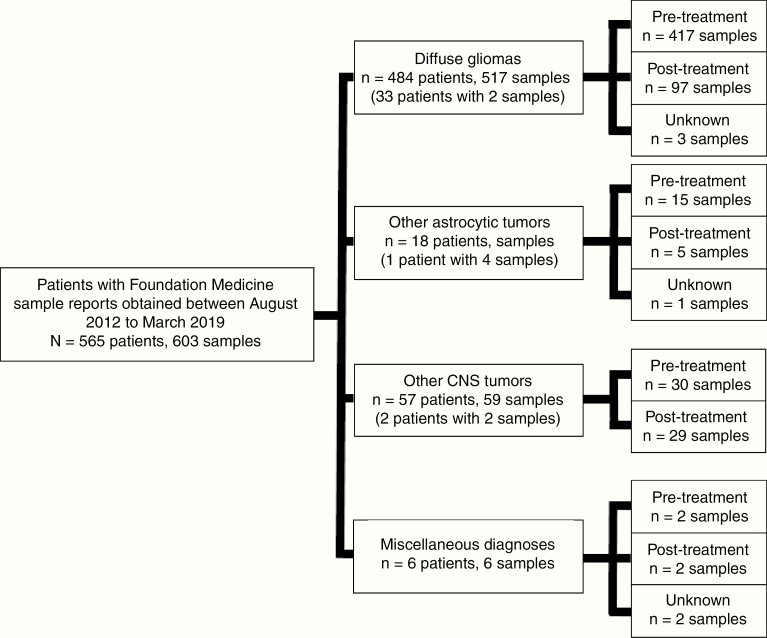
Summary of the initial diagnoses for 565 distinct patients and their 603 Foundation Medicine (FM) samples. There were thirty-six patients who had more than one sample analyzed by FM. These samples were grouped by the treatment status of patient samples.

We identified 603 samples that were sent for FM testing by UCLA neuro-oncologists, which corresponded to 565 distinct patients. Among these 565 patients, 484 were initially diagnosed with diffuse gliomas, 18 with other astrocytic tumors, 57 with other CNS tumors, and six with miscellaneous diagnoses (Figure 1 and Supplementary Table S1). Five hundred and twenty-nine patients received testing on a single sample, 35 patients received testing on two samples, and 1 patient received testing on four samples. The initial diagnoses among the entire cohort, treatment status of each analyzed sample, and types of FM assays utilized were summarized (Supplementary Table S1 and Supplementary Methods).

### Concordance Between Results of Standard Clinical Methods and Foundation Medicine

**Figure 2.**
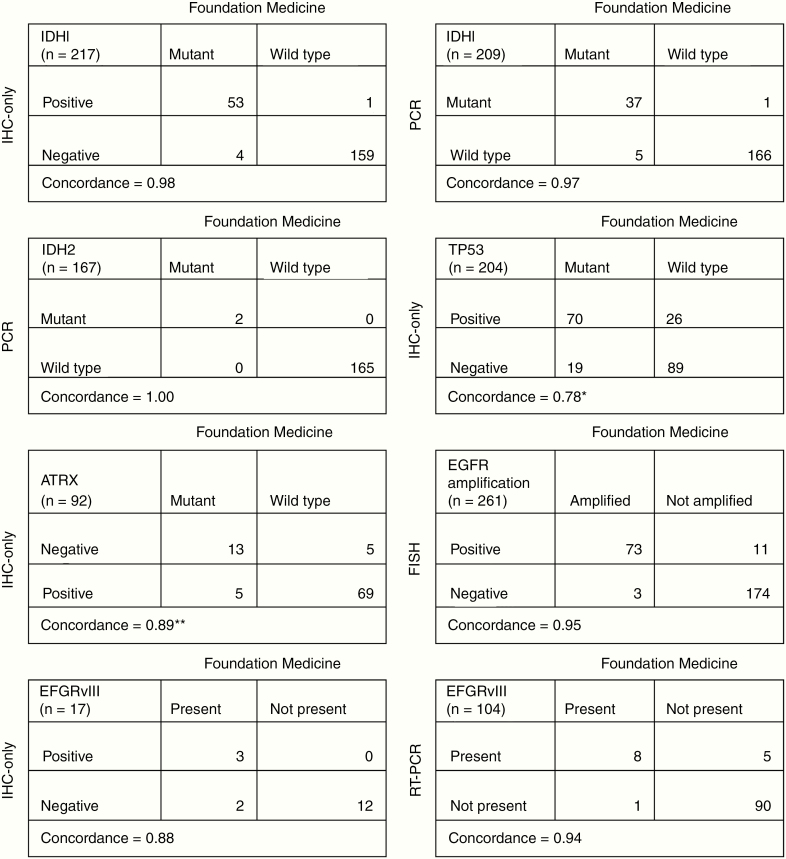
Concordance of molecular testing results between standard clinical methods and Foundation Medicine. *One *TP53* wild-type PCR case was concordant. **3 *ATRX* PCR wild types and 1 PCR mutant were concordant. IHC, Immunohistochemistry; PCR, Polymerase chain reaction sequencing; FISH, Fluorescent in situ hybridization; FM, Foundation Medicine assay method.

The concordance between the results of standard clinical methods and of FM was determined for mutations found in *IDH1/2*, *TP53*, *ATRX,* and *EGFR* (Figure 2). Of the 426 samples that were clinically tested at UCLA for *IDH1* mutations via IHC and PCR, FM identified 99 mutated samples, 87 of which had R132H mutations. In the remaining 12 cases, less common R132 mutations were also identified (R132C, R132S, and R132G) along with other noncanonical mutations (T311I, F32V, D38N, S137F, K243E, and R222C). FM was concordant with 98% of 217 IHC-tested cases, where a positive result indicated an *IDH1* mutation, and 97% of 209 PCR tested cases. Of the 167 samples clinically tested for *IDH2* mutations via PCR, FM only detected two mutated samples exhibiting either an R172K or R172G mutation. All 167 cases were concordant.

In the four discordant *IDH1* IHC-negative cases, FM detected a non-R132H mutation in two cases, which were both R132C mutations. In the third case, different tumor blocks were used between IHC and FM while in the fourth case, the same tumor block was used for both methods. FM always reported an R132H mutation whenever IHC came up positive for an *IDH1* mutation, except in one discordant IHC-positive case. However, for this case, the FM report stated that the sample failed to meet minimum performance standards for comprehensive detection of mutations. The report identified mutations in 12 other genes but did not report any mutations in *IDH1*. For the six (five wild type and one mutant) PCR discordant cases, different tumor blocks were used in the mutant case and in two wild-type cases. For the other three wild-type cases, FM detected the noncanonical T311I, D38N, and F32V mutations.

Of the 204 samples clinically tested for *TP53* mutations via IHC, FM detected 89 mutated samples. Thirty mutated samples contained nonmissense mutations, including deletions, frameshifts, intron truncations, and splice site mutations. FM was concordant with 78% of 204 IHC-tested cases. In 14 of the 19 discordant IHC-negative cases, FM detected nonmissense mutations. In three of the remaining five cases where FM detected R273C, M237I, and S366A mutations, different tumor blocks were used between IHC and FM. In the last two cases where FM detected R273C and H179Q mutations, the same tumor block was used by both methods. In the 26 discordant IHC-positive cases, different tumor blocks were used by both methods in only two cases.

Of the 92 samples clinically tested for *ATRX* mutations via IHC, FM detected 18 mutated samples. Fourteen of the mutated samples had nonmissense mutations, including deletions, frameshifts, insertions, splice site mutations, and bi-allelic loss of *ATRX*. FM was concordant with 89% of 92 IHC-tested cases. In two of the five discordant IHC-positive cases where FM detected nonmissense mutations (a splice site alteration (splice site 54492A > G) and bi-allelic loss of *ATRX*), different tumor blocks were used by IHC and FM. In the last three cases where FM detected missense mutations (N1187K, R2111P, and G446V), the same tumor block was used by both methods. In the five discordant IHC-positive cases, different tumor blocks were used by both methods in all but one.

Of the 261 samples tested for *EGFR* FISH samples, FM detected amplification in 84 cases. FM was concordant with 95% of 261 tested cases. Only in four of the 11 discordant FISH-detected *EGFR* amplified cases were different tumor blocks used by FISH and FM. In all three discordant FISH-detected nonamplified cases, different tumor blocks were used by both methods. Of the 121 EGFRvIII tested samples via IHC and RT-PCR, FM detected EGFRvIII in 12 cases. FM was concordant with 88% of the 17 IHC-tested cases and 94% of 104 RT-PCR cases. In all the discordant IHC and RT-PCR cases, different tumor blocks were used in only one RT-PCR case.

### Diagnostic Differentiation of Oligodendroglioma Versus Astrocytoma Using Foundation Medicine

To assess the ability of the mutations reported by FM to accurately characterize an oligodendroglioma diagnosis, correlations between 1p19q codeletion status and *hTERT* promoter mutations, in addition to other oligodendroglial diagnostic markers (*CIC* and FUBP1 mutations) and astrocytic markers (*ATRX* and *TP53* mutations), were described in 66 pretreatment *IDH* mutated samples that had 1p19q testing. Available *EGFR* amplification and *MGMT* methylation testing results were also included (Supplementary Table S2). Of the 66 samples, 20 *IDH* mutant samples had 1p19q codeletion and 46 had 1p19q retention. Of the 20 1p19q codeleted samples, 17 (85%) also had *hTERT* promoter mutations. Of the 46 1p19q retained samples, only 2 (4.3%) had *hTERT* promoter mutations (Table 1). Of the 17 *hTERT* promoter mutated, 1p19q codeleted samples, 10 samples had *CIC* and/or *FUBP1* mutations while another 6 had neither *ATRX* nor *TP53* mutations. The remaining *hTERT* promoter mutated, 1p19q codeleted sample, Sample 13, had a *TP53* mutation and was considered 1p19q codeleted at a threshold of 30% of tumor cells exhibiting 1p19q codeletion by a pathology report from an outside institution (Supplementary Table S2).

**Table 1.** *hTERT* promoter mutations and 1p19q codeletion status in *IDH* mutant glioma patients (*N* = 66)

	*hTERT* promoter mutation detected: # (% of *n*)	*hTERT* promoter mutation not detected: # (% of *n*)
Positive for 1p19q codeletion (*n* = 20)	17 (85.0%)	3 (15.0%)
Negative for 1p19q codeletion (*n* = 46)	2 (4.3%)	44 (95.7%)

One of the two *hTERT* promoter mutated, 1p19q retained samples, Sample 21, was tested for 1p19q codeletion at UCLA. 50% of Sample 21’s cells were 19q deleted but only 30% of its cells were 1p deleted, which did not meet the UCLA threshold for 1p19q codeletion. However, Sample 21 also had a *FUBP1* mutation and moderate oligodendroglial differentiation. The other sample, Sample 22, had a *TP53* mutation and did not have quantitative data for its retained 1p19q codeletion status. For the three hTERT promoter wild type, 1p19q codeleted samples (Sample 18, 19, and 20), Sample 18 had a *CIC* mutation with rare oligodendroglial differentiation while Sample 19 had *FUBP1* and *CIC* mutations with major oligodendroglial differentiation. Sample 20, which was considered 1p19q codeleted at a threshold of 30% of tumor cells by an outside pathology report, had *ATRX* and *TP53* mutations. One other notable sample, Sample 47, which was an *hTERT* promoter wild type, 1p19q retained sample, had *CIC*, *ATRX*, and *TP53* mutations.

### Intrapatient Tumor Heterogeneity in Patients with Multiple Foundation Medicine Tests

**Figure 3.**
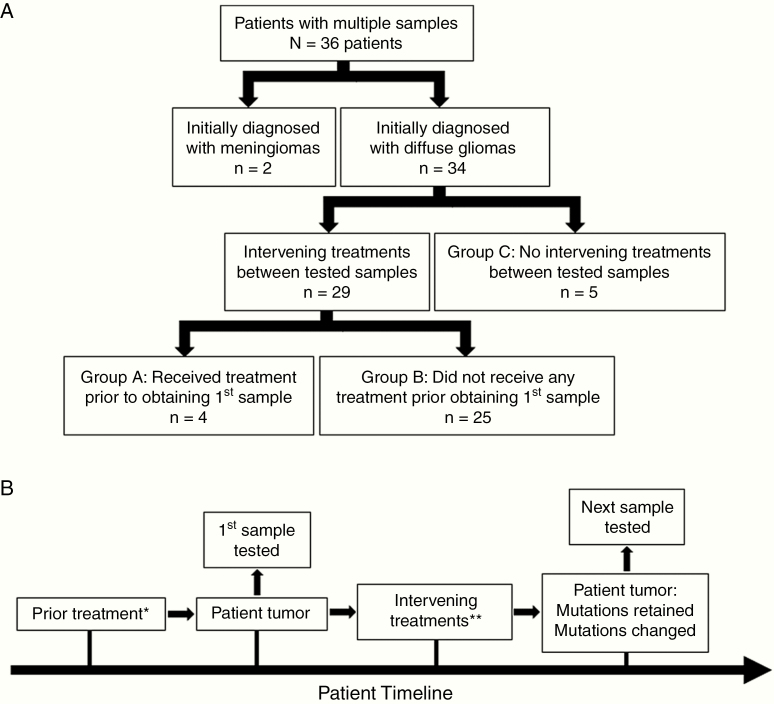
(**A**) Thirty-six patients had multiple samples analyzed by Foundation Medicine (FM) and were grouped according to their initial diagnoses and treatment status. (**B**) Timeline for patients with multiple FM samples. *Prior treatments consisted of temozolomide and/or radiation. **Intervening treatments primarily consisted of temozolomide, radiation, and other chemotherapies.

Of the 36 patients who received testing for more than one sample, 34 were initially diagnosed with diffuse gliomas (Figure 3 and Supplementary Table S3). These 34 patients were divided into those with and without intervening treatments (typically temozolomide, radiation, and an additional chemotherapy) between tested samples. The former group was further divided into patients who received treatment prior to testing their first sample (Group A, *n* = 4) (those who only had post-treatment samples) and patients who did not receive any prior treatment (Group B, *n* = 29) (those who had one pretreatment sample). The group without intervening treatments was designated Group C (*n* = 5). Commonly mutated genes in Group A were *ARID1A, ARID1B*, *ATRX*, *IDH1*, and *TP53* and those in Group B were *CDKN2A*, *CDKN2B*, *EGFR*, *PTEN*, and *hTERT* promoter (Supplementary Table S4). For *EGFR*, there was a multitude of Group B patients that had losses (*n* = 9) and gains (*n* = 8) in mutations. There were also those who later developed bi-allelic losses in *CDKN2A* (*n* = 4) and in *CDKN2B* (*n* = 4). The R132H *IDH1* mutation in all four patients in Group A was retained while there was significant number of Group B patients who retained various mutations, such as bi-allelic losses in *CDKN2A* (*n* = 9), bi-allelic losses in *CDKN2B* (*n* = 11), *EGFR* amplification (*n* = 9), and *hTERT* promoter mutations (*n* = 15).

### Mutations of Unknown Significance Predicting Patient Outcome in GBM

**Figure 4.**
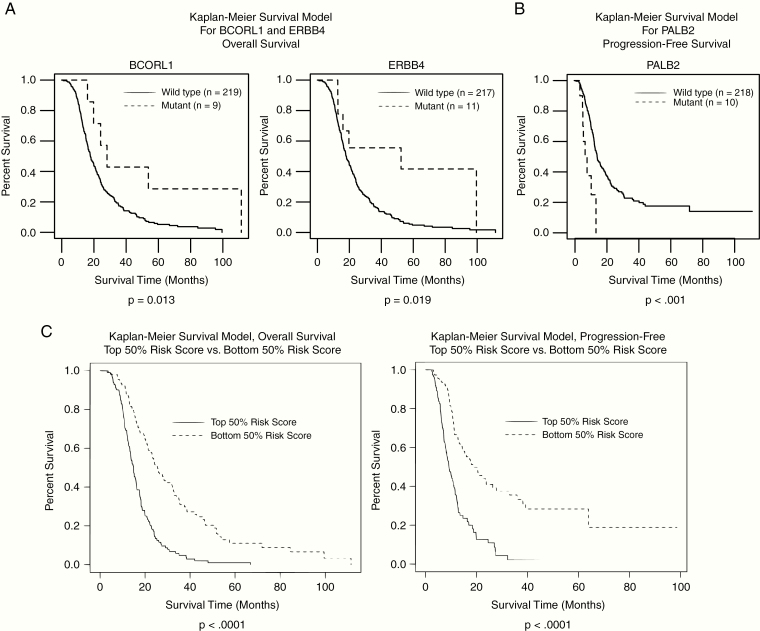
(**A**) Mutations in *BCORL1* and *ERBB4* were identified to contribute to overall survival in this cohort of 228 primary glioblastoma patients by Kaplan–Meier analysis and by a Cox proportional hazards multivariate model. (**B**) Mutations in *PALB2* were identified to contribute to progression-free survival in this cohort of 228 primary glioblastoma patients by Kaplan–Meier analysis and by a Cox proportional hazards multivariate model. (**C**) According to selected mutations, patients with lower risk scores were more likely to have longer overall survival and progression-free survival times. *p* Values less than .05 were considered statistically significant.

Mutations detected by FM that are currently of unknown significance in the context of CNS tumors were explored for their ability to predict patient outcome in primary *IDH* wild-type GBM patients. Out of the 261 who were primary *IDH* wild-type GBM patients, 228 had complete data for all five important clinical covariates: sex, age, EOR, KPS, and *MGMT* methylation status (Supplementary Table S5). Using the elastic net regularized Cox regression, mutations in eight genes detected by FM were identified as important for OS while nine were identified as important for PFS, with six similar mutations overlapping the OS and PFS mutation groups (Table 2). Out of the eight types of mutations identified as contributing to OS, mutations in *BCORL1* (hazard ratio 0.37; 95% confidence interval [CI] 0.14, 1.00; *p* = .049) and *ERBB4* (hazard ratio 0.36; 95% CI 0.14, 0.92; *p* = .033) were shown to be statistically significant by the Cox proportional hazards multivariate model. Out of the nine types of mutations identified as contributing to PFS, only mutations in *PALB2* (hazard ratio 2.49; 95% CI 1.12, 5.52; *p* = .025) were shown to be statistically significant by the multivariate model (Table 2). Kaplan–Meier curves for the mutations that were statistically significant by multivariate analysis are shown in Figure 4.

**Table 2.** Overall survival and progression-free survival cox proportional hazards multivariate models and potential predictive power of the selected mutations

Overall survival				Progression-free survival			
Clinical covariates	Hazard ratio	95% CI	*p-* value	Clinical covariates	Hazard ratio	95% CI	*p-* value
Age at diagnosis	1.02	[1.00, 1.03]	.016	Age at diagnosis	1.01	[1.00, 1.03]	.085
KPS	0.97	[0.95, 0.99]	<.001	KPS	0.98	[0.96, 1.00]	.053
EOR1	1.25	[0.76, 2.06]	.383	EOR1	0.82	[0.46, 1.46]	.493
EOR2	0.98	[0.58, 1.66]	.950	EOR2	0.53	[0.29, 0.99]	.045
*MGMT*	0.47	[0.33, 0.66]	<.001	MGMT	0.43	[0.28, 0.66]	<.001
Sex	1.11	[0.82, 1.51]	.506	Sex	0.9	[0.63, 1.28]	.551
**Selected mutations**							
*BCORL1*	0.37	[0.14, 1.00]	.049	*BCORL1*	0.58	[0.21, 1.61]	.300
*CBL*	0.87	[0.33, 2.32]	.786	*CDK6*	0.28	[0.04, 2.04]	.210
*CDK6*	0.55	[0.17, 1.73]	.304	*CREBBP*	0.92	[0.40, 2.11]	.836
*DOT1L*	0.76	[0.26, 2.27]	.628	*DOT1L*	0.95	[0.28, 3.23]	.940
*ERBB4*	0.36	[0.14, 0.92]	.033	*ERBB4*	0.41	[0.15, 1.16]	.093
*IGF1R*	0.59	[0.27, 1.28]	.183	*IGF1R*	0.74	[0.29, 1.86]	.523
*NF1*	0.78	[0.52, 1.18]	.241	*PALB2*	2.49	[1.12, 5.52]	.025
*RB1*	1.05	[0.67, 1.65]	.830	*RET*	0.45	[0.14, 1.47]	.187
				*SPEN*	0.56	[0.29, 1.08]	.083
**R-squared statistics describing the explanatory power on overall survival and progression-free survival of selected mutations**							
Selected mutations included?	No		Yes	Selected mutations included?	No		Yes
R-square value	0.09		0.25	R-square value	0.07		0.32
**Comparison of risk scores by Kaplan-Meier analysis**							
Risk scores	Median (months)	95% CI	*p*-value	Risk scores	Median (months)	95% CI	*p*-value
Top 50%	14.9	[13.3, 16.3]	<.001	Top 50%	10.0	[8.6, 11.6]	<.001
Bottom 50%	25.5	[21.9, 32.6]		Bottom 50%	18.6	[13.2, 32.9]	

CI, confidence interval; EOR1, 10–90% extent of tumor resection; EOR2, >90% extent of tumor resection; KPS, Karnofsky performance score.

To evaluate the potential predictive power of the selected mutations for OS and PFS, R-squared statistics for a model with only the five clinical covariates and another model including both the selected mutations and the clinical covariates were calculated for both OS and PFS settings. It was observed that adding the selected mutations to the clinical covariates increased the R-squared statistic from 0.16 to 0.34 in the OS setting and from 0.09 to 0.25 in the PFS setting, representing substantially added explanatory power. It is evident that patients with a higher risk score based on the selected mutations are associated with much shorter OS (median OS 14.9 months; 95% CI 13.3, 16.3 vs 25.5 months; 95% CI 21.9, 32.6; *p* < .001) and PFS (median PFS 10 months; 95% CI 8.6, 11.6 vs 18.6 months; 95% CI 13.2, 32.9; *p* < .001; Figure 4 and Table 2).

## Discussion

For the first objective of this study, the concordance between the results of standard clinical methods and of FM was assessed to confirm the reliability of the FM targeted NGS platform. For detecting *IDH* mutations, FM seemed to be quite reliable. Five of the 11 discordant cases were comprised of situations where standard clinical methods could not detect mutations that FM could, such as an R132C in discordant IHC cases and other noncanonical mutations in discordant PCR cases. For the last six cases, discordance could be explained by factors such as technical failure or different tumor blocks being used between IHC/PCR and FM, which may cause discordance due to possible spatial mutational differences or sampling error.^12,13^

For *TP53* mutations, concordance with IHC was much lower, perhaps due to the heterogeneity in *TP53* mutations. It is known that nonmissense mutations in *TP53* can lead to IHC-negative results due to these mutations potentially preventing the expression of the p53 protein that IHC directly detects.^19,20^ This may be the reason why out of the 30 cases where nonmissense mutations were detected by FM, 14 were IHC negative. Among the other 5 IHC-negative and 26 IHC-positive discordant cases, some discordance may have arisen due to technical and sampling errors. Despite concordance being lower compared to *IDH*, the numbers in this study are quite similar to other situations when IHC and PCR are used to detect *TP53* mutations. In one study comparing the results between IHC and PCR to detect only *TP53* missense mutations in 61 low-grade gliomas, IHC was reported to have a sensitivity of 92% and specificity of 79% when comparing to PCR.^20^ When removing all 30 nonmissense mutated cases from our analysis, the calculated sensitivity and specificity values are 91.5% and 77.4% when comparing IHC to FM, which are similar to the study previously mentioned.

For *ATRX* mutations, EGFRvIII, and *EGFR* amplification, concordance was relatively high. Concordance for *ATRX* (89%) was similar to numbers reported in the literature, where one study reported a concordance of 84.6% in 78 tumor samples when comparing IHC to whole-exome sequencing (WES), which is a more comprehensive form of NGS.^21^ Like in *ATRX*, the concordance for *EGFR* amplification (95%) was similar to that in the literature, where a 92% concordance was reported in a study that was comparing FISH to WES. This study also reported several cases that were FISH positive but WES negative. FISH-positive cases with low number of positive cells may not be considered amplified by such methods as WES and targeted NGS, which may employ a more stringent copy number normalization upon the entire tumor that can cause some samples to not meet NGS-positive amplification thresholds.^22^

For the second objective, mutations detected by FM were assessed for their ability to predict the presence of 1p19q codeletion in *IDH* mutant glioma patients. In this study, *hTERT* promoter mutations were detected in 85% of 1p19q codeleted, *IDH* mutant glioma samples or WHO 2016 classified oligodendrogliomas, which confirms previous studies that have observed a high frequency of *hTERT* promoter mutations in oligodendroglial tumors.^9,11^ This suggests that in most *IDH* mutant gliomas, the presence of *hTERT* promoter mutations could perform as a surrogate for 1p19q codeletion when diagnosing oligodendrogliomas. Combined with *hTERT* promoter mutations, the presence of *CIC* and *FUBP1* mutations and oligodendroglial histopathological features and the absence of *ATRX* and *TP53* mutations may more strongly indicate whether a tumor is 1p19q codeleted as seen in the many *hTERT* promoter mutated, 1p19q codeleted patients of this study.^23^ This “rule” holds in the two other *hTERT* mutated samples. Sample 21 had an *FUBP1* mutation, had moderate oligodendroglial differentiation, and could arguably be considered 1p19q codeleted. Sample 22 only had a *TP53* mutation, was absent of *FUBP1* and *CIC* mutations, and was 1p19q retained. An exception was Sample 13, which was *hTERT* promoter mutated, 1p19q codeleted but had a *TP53* mutation and no other oligodendroglial features.

In addition, situations such as Sample 18 and 19, which appeared to be oligodendrogliomas, may demonstrate how *FUBP1* and *CIC* mutations and histopathological features without the *hTERT* promoter mutation could potentially predict the presence of 1p19q codeletion. Sample 47, the only *hTERT* promoter wild type, 1p19q retained sample that had a *CIC* or *FUPB1* mutation, seems to follow this “rule” as it had both *ATRX* and *TP53* mutations, which are clear astrocytic markers, and no mention of oligodendroglial features in the patient’s pathology report. An exception is Sample 20, another *hTERT* promoter wild type, dual *ATRX* and *TP53* mutated sample that was considered to be 1p19q codeleted at a lower threshold.

For the third objective, patients with multiple samples that were tested by FM were studied to describe intrapatient tumor heterogeneity. This was seen especially in *EGFR* in Group B patients, where there were numerous cases of temporal changes in mutations potentially associated by intervening treatments, such as temozolomide. Even within samples that did not undergo treatment and in samples of the same tumor block (Group C), there was still mutational heterogeneity. One notable example in Group C was Patient 34 who appeared to have gained a mutation in *ATRX,* a key mutation involved in diagnosing astrocytic tumors (Supplementary Table S3). Temporal retention in mutations, such as *IDH1*, *hTERT* promoter, and *CDKN2A/B* bi-allelic loss, in Group A and B was expected as these are considered to be driver mutations that may provide proliferative advantage under treatment.^12^

For the last objective, mutations detected by FM that are of unknown significance in the context of GBM were assessed for their ability to predict patient outcome. Multivariate analysis showed that mutations in *BCORL1* and *ERBB4* were predictors of OS and that mutations in *PALB2* are predictors of PFS. All three of these genetic mutations are quite rare in the context of GBM according to this study and the GBM TCGA dataset, where *BCORL1, ERBB4,* and *PALB2* mutations affected 2.54%, 2.04%, and 1.78% of 393 cases, respectively.^24^*BCORL1,* an X chromosome gene that encodes for a corepressor of E-cadherin, has been implicated in melanoma and acute myeloid leukemia.^25,26^ In addition, an increased expression in *BCORL1* has been shown to lead to lower OS and PFS in hepatocellular carcinoma.^27^ Lost expression of E-cadherin, an adhesive molecule of the epithelium, has been shown to contribute to tumor metastasis by facilitating epithelial-mesenchymal transition, while an upregulation of E-cadherin expression and adhesive function has been shown to impede tumor expansion.^27–29^ The nine *BCORL1-*mutated patients had better OS than nonmutated patients, suggesting that their mutations could be deactivating mutations in *BCORL1* that would decrease repression of E-cadherin.

The 11 patients with mutations in *ERBB4*, also had better OS than their wild-type counterparts. ERBB4, similar to EGFR, is a transmembrane receptor tyrosine kinase that is composed of different variants that are expressed in a tissue-specific manner.^30^ ERBB4 variants with JM-a, a tumor necrosis factor-alpha converting enzyme cleavable extracellular domain, and with an intracellular domain CYT-2 were shown to predominantly be expressed in GBM tissue and activated via phosphorylation in GBM patients with lower survival.^30^ In another study on non-small cell lung cancer, inhibition of ERBB4 signaling was shown to slow tumor progression.^31^ It may be the case that the mutations found in these 11 patients could be deactivating mutations that could inhibit ERBB4 signaling similarly in GBM.

The 10 patients with mutations in *PALB2* had worse PFS compared to their counterparts. PALB2 is involved in DNA damage response and repair together with BRCA1/2, and its mutations have been shown to increase risk for breast and pancreatic cancers.^32,33^ Treatments, such as PARP inhibitors and platinum agents, have been shown to improve clinical outcomes in *PALB2* mutated breast and pancreatic patients.^33,34^ Potentially deactivating mutations in *PALB2* may be the reason for the higher risk of progression within these patients. While there has not been much in the literature specifically for *BCORL1* or *ERBB4* as potential therapeutic targets in the context of GBM, there have been suggestions for studying a FDA-approved olapirib, a PARP inhibitor that has been shown to be brain permeable, to be used for *PALB2* mutated GBM patients.^34^ The significance of these genetic mutations in GBM patient outcome may warrant further studies on specific mutations within these genes and related genes in similar pathways in the context of gliomas.

As a retrospective, descriptive study, there were limitations in the accuracy of initial diagnoses, histopathological features, molecular and genetic testing results, and other clinical covariates as these were all recorded from pathology reports. In addition, some reports were done at institutions other than UCLA and may suffer from differences in institutional protocol as was seen with the 1p19q codeletion data. There was no central review of IHC or FISH results. In addition, the raw FM data files were not available to determine how thresholding may influence our results.

## Conclusion

This retrospective, descriptive study suggests that targeted next-generation sequencing platforms, such as Foundation Medicine, enhance the molecular characterization of CNS tumors in the clinical setting.

## Supplementary Material

vdaa009_suppl_Supplemental_Methods

vdaa009_suppl_Supplemental_Table_S1

vdaa009_suppl_Supplemental_Table_S2

vdaa009_suppl_Supplemental_Table_S3

vdaa009_suppl_Supplemental_Table_S4

vdaa009_suppl_Supplemental_Table_S5

## Notes

**Conflict of interest statement.** T.F.C. has received personal fees from Pfizer, Tocagen, Roche, Novocure, Nektar, VBL, Abbvie, Upshire Smith, Notable Labs, Oxigene, NewGen, Agios, Cortice, MedQia, PRoNai, Wellcome, Merck, Insys, Human Longevity, Sunovion, Boston Biomedical, Alexion, Novogen, outside the submitted work. T.F.C. is a board member of the Global Coalition for Adaptive Research 501c3 and the PI for GBM Agile. T.F.C. has stock options for Notable Labs. A.L. has received honoraria from Merck, Genentech, Abbvie, and Optune. P.L.N. has received grants from Genentech/Roche.
