# Supplementary material for: Targeted next-generation sequencing of 565 neuro-oncology patients at UCLA: A single-institution experience
Source: Neurooncol Adv. 2020 Jan 29;2(1):vdaa009. doi: 10.1093/noajnl/vdaa009 (PMC7034640; doi:10.1093/noajnl/vdaa009)
Supplement: vdaa009_suppl_Supplemental_Methods [file vdaa009_suppl_supplemental_methods.docx]

**Supplemental Methods:**

Foundation Medicine utilizes a hybrid-capture, NGS method.^1^ Patients included in this study received one of the various FM’s genomic profiling assays: FoundationOne, FoundationOne Cdx, FoundationOneHeme, and FoundationACT.^1,2,3^ All three profiling assays utilize FM’s hybrid-capture, NGS method and are primarily differentiated by the type of input sample (e.g. FFPE tumor or peripheral whole blood sample) and the number of targeted mutation they are designed to detect. The FoundationOne assay utilizes FFPE tumor samples and two versions of the assay were performed, one version (FoundationOne vI) that could detect mutations in 236 genes and intron rearrangements in 19 genes and a second more comprehensive version (FoundationOne vII) that could detect mutations in 315 genes and intron rearrangements in 28 genes. The FoundationOne Cdx assay, a FDA-approved version of the older FoundationOne assay, also utilizes FFPE tumor samples and is designed to detect 324 genes and intron rearrangements in 36 genes and quantify microsatellite instability and tumor mutational burden. The FoundationOneHeme assay, which is mainly used for hematologic diseases, utilizes FFPE tumor, bone marrow aspirate, and peripheral whole blood samples and can detect mutations in 405 genes, intron rearrangements in 31 genes, and microsatellite instability and tumor mutational burden (TMB). The FoundationACT assay, a minimally invasive liquid biopsy test, utilizes peripheral whole blood and can detect mutations in 27 genes, mutations in exons of an additional 34 genes, and intron rearrangements in six genes.

**References**

1. Frampton GM, Fichtenholtz A, Otto GA, et al. Development and validation of a clinical cancer genomic profiling test based on massively parallel DNA sequencing. Nat Biotechnol. 2013; 31(11): 1023-1031.
2. He J, Abdel-Wahab O, Nahas MK, et al. Integrated genomic DNA/RNA profiling of hematologic malignancies in the clinical setting. Blood. 2016; 127(24): 3004-3014.
3. Stephens PJ, Clark T, Kennedy M, et al. Analytic validation of a clinical circulating tumor DNA assay for patients with solid tumors. Ann Oncol. 2016; 27(suppl_6).
