# Supplementary material for: Targeted next-generation sequencing of 565 neuro-oncology patients at UCLA: A single-institution experience
Source: Neurooncol Adv. 2020 Jan 29;2(1):vdaa009. doi: 10.1093/noajnl/vdaa009 (PMC7034640; doi:10.1093/noajnl/vdaa009)
Supplement: vdaa009_suppl_Supplemental_Table_S1 [file vdaa009_suppl_supplemental_table_s1.docx]

| **Supplementary Table S1. Initial diagnoses of patients, treatment statuses of their Foundation Medicine samples, and types of Foundation Medicine assays utilized to analyze each sample** | | | | | | | | | |
| --- | --- | --- | --- | --- | --- | --- | --- | --- | --- |
|  | **Initial diagnosis** | **Patient total**  **n (% of 565)** | **Treatment status** | **Sample total** | **FoundationOne vI***** | **FoundationOne vII***** | **FoundationOne Cdx***** | **FoundationOneHeme***** | **Foundation**  **ACT***** |
|  | Glioblastoma, Grade IV* | 331 (58.6%) | pre** | 298 | 19 | 233 | 44 | 0 | 1 |
|  |  |  | post** | 49 | 3 | 36 | 10 | 0 | 0 |
| Diffuse Gliomas |  |  | unknown | 3 | 0 | 3 | 0 | 0 | 0 |
|  | Gliosarcoma, Grade IV* | 5 (0.9%) | pre | 3 | 0 | 2 | 1 | 0 | 0 |
|  |  |  | post** | 3 | 1 | 2 | 0 | 0 | 0 |
|  | Diffuse midline glioma, Grade IV | 2 (0.3%) | pre | 2 | 0 | 1 | 1 | 0 | 0 |
|  | Anaplastic astrocytoma, Grade III* | 55 (9.7%) | pre** | 48 | 3 | 39 | 6 | 0 | 0 |
|  |  |  | post** | 10 | 2 | 8 | 0 | 0 | 0 |
|  | Anaplastic oligoastroctyoma, Grade III* | 6 (1.1%) | pre | 3 | 0 | 1 | 2 | 0 | 0 |
|  |  |  | post** | 5 | 1 | 3 | 0 | 1 | 0 |
|  | Anaplastic oligodendroglioma, Grade III* | 14 (2.5%) | pre | 10 | 0 | 9 | 1 | 0 | 0 |
|  |  |  | post** | 6 | 0 | 6 | 0 | 0 | 0 |
|  | Astrocytoma, Grade II* | 29 (5.1%) | pre** | 24 | 1 | 21 | 2 | 0 | 0 |
|  |  |  | post** | 8 | 0 | 8 | 0 | 0 | 0 |
|  | Oligoastrocytoma, Grade II* | 25 (4.4%) | pre** | 17 | 1 | 14 | 2 | 0 | 0 |
|  |  |  | post** | 10 | 2 | 7 | 1 | 0 | 0 |
|  | Oligodendroglioma, Grade II* | 17 (3.0%) | pre | 12 | 1 | 10 | 1 | 0 | 0 |
|  |  |  | post | 6 | 0 | 4 | 2 | 0 | 0 |
| Other Astrocytic Tumors | Anaplastic pilocytic astrocytoma, Grade II | 2 (0.4%) | pre | 2 | 0 | 2 | 0 | 0 | 0 |
|  | Pilocytic astrocytoma, Grade I/low grade | 12 (2.1%) | pre | 9 | 0 | 8 | 1 | 0 | 0 |
|  |  |  | post | 2 | 0 | 2 | 0 | 0 | 0 |
|  |  |  | unknown | 1 | 0 | 1 | 0 | 0 | 0 |
|  | Subependymal giant cell astrocytoma, Grade I* | 1 (0.2%) | pre | 1 | 1 | 0 | 0 | 0 | 0 |
|  |  |  | post** | 3 | 1 | 2 | 0 | 0 | 0 |
|  | Anaplastic pleomorphic xanthoastrocytoma, Grade III | 1 (0.2%) | pre | 1 | 0 | 1 | 0 | 0 | 0 |
|  | Pleomorphic xanthoastrocytoma, Grade II | 1 (0.2%) | pre | 1 | 0 | 1 | 0 | 0 | 0 |
|  | Pilomyxoid astrocytoma, Grade II | 1 (0.2%) | pre | 1 | 0 | 1 | 0 | 0 | 0 |
|  | Anaplastic ependymoma, Grade III | 4 (0.7%) | pre | 3 | 0 | 3 | 0 | 0 | 0 |
| Other CNS Tumors |  |  | post | 1 | 0 | 1 | 0 | 0 | 0 |
|  | Anaplastic myxopapillary ependymoma, Grade III | 1 (0.2%) | post | 1 | 0 | 1 | 0 | 0 | 0 |
|  | Ependymoma, Grade II | 3 (0.5%) | pre | 2 | 0 | 2 | 0 | 0 | 0 |
|  |  |  | post | 1 | 0 | 1 | 0 | 0 | 0 |
|  | Diffuse leptomeningeal glioneuronal tumor, Grade III | 1 (0.2%) | pre | 1 | 0 | 1 | 0 | 0 | 0 |
| Other CNS Tumors | Extraventricular neurocytoma, Grade II | 1 (0.2%) | pre | 1 | 0 | 1 | 0 | 0 | 0 |
|  | Ganglioglioma grade I/II | 3 (0.5%) | pre | 1 | 0 | 1 | 0 | 0 | 0 |
|  |  |  | post | 2 | 0 | 2 | 0 | 0 | 0 |
|  | Suprasellar/3rd ventricle glioneuronal tumor | 1 (0.2%) | pre | 1 | 0 | 1 | 0 | 0 | 0 |
|  | Pineal parenchymal tumor of intermediate differentiation (PPTID), Grade III | 1 (0.2%) | pre | 1 | 0 | 1 | 0 | 0 | 0 |
|  | Pineal pilocytic astrocytoma, Grade III | 1 (0.2%) | pre | 1 | 0 | 1 | 0 | 0 | 0 |
|  | Pineocytoma, Grade I | 1 (0.2%) | post | 1 | 0 | 1 | 0 | 0 | 0 |
|  | Papillary tumor of the pineal region, Grade III | 1 (0.2%) | post | 1 | 0 | 1 | 0 | 0 | 0 |
|  | Medulloblastoma, Grade IV | 7 (1.2%) | pre | 3 | 1 | 1 | 1 | 0 | 0 |
|  |  |  | post | 4 | 2 | 1 | 1 | 0 | 0 |
|  | CNS embryonal tumor, Grade IV | 1 (0.2%) | pre | 1 | 0 | 1 | 0 | 0 | 0 |
|  | Esthesioneuroblastoma | 1 (0.2%) | post | 1 | 0 | 1 | 0 | 0 | 0 |
|  | Schwannoma, Grade I | 1 (0.2%) | pre | 1 | 0 | 0 | 1 | 0 | 0 |
|  | Neurofibroma | 1 (0.2%) | pre | 1 | 0 | 1 | 0 | 0 | 0 |
|  | Meningioma, Grade I/II* | 23 (3.9%) | pre | 11 | 0 | 9 | 2 | 0 | 0 |
|  |  |  | post** | 14 | 1 | 11 | 2 | 0 | 0 |
|  | Hemangiopericytoma, Grade II | 2 (0.3%) | pre | 1 | 0 | 0 | 0 | 1 | 0 |
|  |  |  | post | 1 | 0 | 0 | 0 | 1 | 0 |
|  | Craniopharyngioma | 2 (0.3%) | post | 2 | 0 | 1 | 1 | 0 | 0 |
|  | Optic nerve glioma, Grade II | 1 (0.2%) | pre | 1 | 0 | 1 | 0 | 0 | 0 |
| Miscellaneous Diagnoses | Temporal sclerosis, Grade II | 1 (0.2%) | pre | 1 | 0 | 1 | 0 | 0 | 0 |
|  | Type IIb cortical dysplasia | 1 (0.2%) | unknown | 1 | 0 | 1 | 0 | 0 | 0 |
|  | Atypical gliosis | 1 (0.2%) | pre | 1 | 0 | 1 | 0 | 0 | 0 |
|  | Metastatic carcinoma | 3 (0.5%) | post | 2 | 0 | 1 | 1 | 0 | 0 |
|  |  |  | unknown | 1 | 0 | 1 | 0 | 0 | 0 |

Treatment status: pre-samples were obtained before patient received any treatment (pre-treatment), post-samples were obtained after patient received treatment (post-treatment), unknown-samples were sample not able to be classified due to unknown treatment status of patient at the time they were obtained.*Patients in this group received Foundation Medicine (FM) testing on additional samples and have more than one FM report available. **Total comprises of additional FM samples available from patients who have more than one FM samples. ***Types of Foundation Medicine assays are described in the Supplemental Methods.
