# Supplementary material for: Targeted next-generation sequencing of 565 neuro-oncology patients at UCLA: A single-institution experience
Source: Neurooncol Adv. 2020 Jan 29;2(1):vdaa009. doi: 10.1093/noajnl/vdaa009 (PMC7034640; doi:10.1093/noajnl/vdaa009)
Supplement: vdaa009_suppl_Supplemental_Table_S2 [file vdaa009_suppl_supplemental_table_s2.docx]

| **Supplemental Table S2: Foundation Medicine tested mutations in *IDH* mutant glioma samples with FISH tested 1p19q co-deletion status (N=66)** | | | | | | | | |
| --- | --- | --- | --- | --- | --- | --- | --- | --- |
| Patient Sample | hTERT promoter  (MUT/WT) | CIC  (MUT/WT) | FUBP1  (MUT/WT) | ATRX  (MUT/WT) | TP53  (MUT/WT) | EGFR amplification  (Yes/No) | MGMT  Methylation*  (M/U/NA) | 1p19q  co-deletion  (Yes/No) |
| 1 | MUT | MUT | MUT | WT | MUT | No | NA | Yes |
| 2 | MUT | WT | WT | WT | WT | No | M | Yes |
| 3 | MUT | MUT | WT | WT | WT | No | NA | Yes |
| 4 | MUT | MUT | WT | WT | WT | No | NA | Yes |
| 5 | MUT | MUT | MUT | WT | MUT | No | M | Yes |
| 6 | MUT | MUT | WT | WT | WT | No | M | Yes |
| 7 | MUT | WT | WT | WT | WT | Yes | M | Yes |
| 8 | MUT | MUT | WT | WT | WT | No | M | Yes |
| 9 | MUT | WT | WT | WT | WT | No | NA | Yes |
| 10 | MUT | MUT | MUT | WT | WT | No | NA | Yes |
| 11 | MUT | MUT | WT | WT | WT | No | M | Yes |
| 12 | MUT | WT | WT | WT | WT | No | NA | Yes |
| 13 | MUT | WT | WT | WT | MUT | No | M | Yes*** |
| 14 | MUT | MUT | WT | MUT | WT | No | M | Yes |
| 15 | MUT | MUT | MUT | WT | WT | No | M | Yes |
| 16 | MUT | WT | WT | WT | WT | No | NA | Yes |
| 17 | MUT | WT | WT | WT | WT | No | M | Yes |
| 18 | WT | MUT | WT | WT | WT | No | U | Yes |
| 19 | WT | MUT | MUT | WT | WT | No | M | Yes |
| 20 | WT | WT | WT | MUT | MUT | No | M | Yes** |
| 21 | MUT | WT | MUT | MUT | WT | No | M | No*** |
| 22 | MUT | WT | WT | WT | MUT | No | M | No |
| 23 | WT | WT | WT | MUT | MUT | No | M | No |
| 24 | WT | WT | WT | MUT | MUT | No | U | No |
| 25 | WT | WT | WT | MUT | MUT | No | U | No |
| 26 | WT | WT | WT | MUT | MUT | No | M | No |
| 27 | WT | WT | WT | MUT | MUT | No | NA | No |
| 28 | WT | WT | WT | MUT | MUT | No | M | No |
| 29 | WT | WT | WT | MUT | MUT | No | NA | No |
| 30 | WT | WT | WT | MUT | MUT | No | NA | No |
| 31 | WT | WT | WT | MUT | MUT | No | M | No |
| 32 | WT | WT | WT | MUT | MUT | No | M | No |
| 33 | WT | WT | WT | MUT | MUT | No | U | No |
| 34 | WT | WT | WT | MUT | MUT | No | M | No |
| 35 | WT | WT | WT | MUT | MUT | No | M | No |
| 36 | WT | WT | WT | MUT | MUT | No | U | No |
| 37 | WT | WT | WT | MUT | MUT | No | M | No |
| 38 | WT | WT | WT | MUT | MUT | No | U | No |
| 39 | WT | WT | WT | MUT | MUT | No | NA | No |
| 40 | WT | WT | WT | MUT | MUT | No | NA | No |
| 41 | WT | WT | WT | MUT | MUT | No | M | No |
| 42 | WT | WT | WT | MUT | MUT | No | U | No |
| 43 | WT | WT | WT | MUT | MUT | No | U | No |
| 44 | WT | WT | WT | MUT | MUT | No | U | No |
| 45 | WT | WT | WT | MUT | MUT | No | U | No |
| 46 | WT | WT | WT | MUT | MUT | No | U | No |
| 47 | WT | MUT | WT | MUT | MUT | No | M | No |
| 48 | WT | WT | WT | WT | WT | No | U | No |
| 49 | WT | WT | WT | MUT | MUT | No | M | No |
| 50 | WT | WT | WT | MUT | MUT | No | U | No |
| 51 | WT | WT | WT | MUT | MUT | No | U | No |
| 52 | WT | WT | WT | MUT | MUT | No | NA | No |
| 53 | WT | WT | WT | MUT | MUT | No | NA | No |
| 54 | WT | WT | WT | MUT | MUT | No | M | No |
| 55 | WT | WT | WT | WT | MUT | No | NA | No |
| 56 | WT | WT | WT | MUT | MUT | No | NA | No |
| 57 | WT | WT | WT | MUT | MUT | No | M | No |
| 58 | WT | WT | WT | MUT | MUT | No | NA | No |
| 59 | WT | WT | WT | MUT | MUT | No | M | No |
| 60 | WT | WT | WT | MUT | MUT | No | U | No |
| 61 | WT | WT | WT | MUT | MUT | No | M | No |
| 62 | WT | WT | WT | MUT | MUT | No | NA | No |
| 63 | WT | WT | WT | MUT | MUT | No | M | No |
| 64 | WT | WT | WT | MUT | MUT | No | NA | No |
| 65 | WT | WT | WT | MUT | MUT | No | M | No |
| 66 | WT | WT | WT | MUT | MUT | No | M | No |

MUT: Mutant, WT: Wild type, M: Methylated, U: Unmethylated, NA: Not available, FISH: fluorescent in situ hybridization.

*Not detected by Foundation Medicine but done by methylation specific real time polymerase chain reaction amplified sequencing.

**1p19q co-deletion was reported when there was only 30% deletion in both 1p and 19q.

***1p19q retention was reported when there was over 50% deletion in 19q and 30% deletion in 1p.
