# Supplementary material for: Targeted next-generation sequencing of 565 neuro-oncology patients at UCLA: A single-institution experience
Source: Neurooncol Adv. 2020 Jan 29;2(1):vdaa009. doi: 10.1093/noajnl/vdaa009 (PMC7034640; doi:10.1093/noajnl/vdaa009)
Supplement: vdaa009_suppl_Supplemental_Table_S3 [file vdaa009_suppl_supplemental_table_s3.docx]

| **Supplementary Table S3. Initial diagnoses of patients who received multiple Foundation Medicine tests, treatment statuses of their Foundation Medicine samples, type of Foundation Medicine assay utilized to analyze each sample, intervening treatments between sample analyses, and retention of mutations and changes in mutations between samples** | | | | | | | | | |
| --- | --- | --- | --- | --- | --- | --- | --- | --- | --- |
| **Patient** | | **Initial diagnosis** | **Malignant transformation?**  **(Updated diagnosis)** | **Type of Foundation Medicine assay utilized to analyze each sample and treatment status of sample** | **Compared FM samples** | **Intervening treatments**  **between sample analysis** | **Mutations retained**  ***Gene*(mutation1, mutation3, mutation3…)** | **Mutations lost**  ***Gene*(mutation1, mutation2, mutation3…)** | **Mutations gained**  ***Gene*(mutation1, mutation2, mutation3…)** |
| Meningiomas | 1 | MENG-Grade I | Yes, MENG-Grade III | 1^st^ FM sample:  FoundationOne vI  (post-treatment)  2^nd^ FM sample:  FoundationOne vII  (post-treatment) | 1^st^-2^nd^ | TEM, BEV, TRAM | *CHEK2*(T476M), *EGFR*(rearrangement), *MDM4*(T113A), *MED12*(R2043H), *NF2*(Q212*), *PRDM1*(V762M) | NA | *BARD1*(P358_S364del), *SPEN*(A2571V) |
|  | 2 | MENG-  Grade II | No | 1^st^ FM sample:  FoundationOne vII  (post-treatment)  2^nd^ FM sample:  FoundationOne Cdx  (post-treatment) | 1^st^-2^nd^ | Proton "pencil" beam, BEV | *ESR1*(H6Y), *NF2*(E445fs*9), | NA | *BAP1*(R163_M165del), *CDK4*(amplification) |
| Group A | 3 | AO | No | 1^st^ FM sample:  FoundationOne vII  (post-treatment)  2^nd^ FM sample:  FoundationOne vII  (post-treatment) | 1^st^-2^nd^ | TEM, RT, VIN, CARB, VOR | *APC*(E1209K), *ARID1B*(Q129_Q130insQ), *ASXL1*(A1199T), *ATM*(H1082R), *ATRX*(R808Q), *AXL*(K657R), *CIC*(R215W),  *IDH1*(R132H), *LRP1B*(E1669fs*12), *FUBP1*(I278fs*12)*, MLL2*(A569_E577del), *NOTCH1*(F357del), *RUNX1*(E138D), *SOCS1*(A49S), *hTERT* promoter(-124C>T) | *CDK8*(N156D), *NOTCH1*(N454del, S715fs*56), *FBXW7*(E113D), *MEN1*(N357S), *SOX2*(amplification) | *ARID1A*(Q1364*, Q2176fs*48), *FGFR3*(E447K), *KEL*(Y250C),  *KIT*(P871L), *MLL3*(N1353fs*21), *NOTCH1*(N947S), *PRKDC*(D3094fs*2), *TSHR*(I44V) |
|  | 4 | AOA | Yes, GBM | 1^st^ FM sample:  FoundationOne vII  (post-treatment)  2^nd^ FM sample:  FoundationOne vII  (post-treatment) | 1^st^-2^nd^ | TEM, LOM | *ARID1B*(C878R), *ATRX*(A238fs*19), *FAT1*(R4208W), *FLT1*(S722del), *IDH1*(R132H), *NOTCH3*(V1062M), *NTRK1*(R6W), *TNFRSF14*(W12C), *TP53*(S127Y) | *BRIP1*(amplification), | *CDKN2A*(bi-allelic loss), *CDKN2B*(bi-allelic loss), *FAM46C*(G242R), *FANCC*(amplification), *GNAQ*(amplification), *MET*(amplification), *NTRK2*(amplification), *PTCH1*(amplification), *SLIT2*(S1223C), *SYK*(amplification) |
|  | 5 | OA | Yes, GBM | 1^st^ FM sample:  FoundationOne vII  (post-treatment)  2^nd^ FM sample:  FoundationOne vII  (post-treatment) | 1^st^-2^nd^ | TEM | *APC*(A426S), *ATRX*(V2189A), *CDH1*(T529A), *FANCG*(P241L), *IDH1*(R132H), TSC1(Q654E), *TP53*(Q167fs*3) | NA | *ABL1*(D1113N), *AKT3*(G308D), *APC*(V47I), *ARID1A*(A1413V, splice site 21621G>A), *ARID1B*(G316E), *ASXL1*(D800N), *ATR*(splice site 76561G>A, A659T, P1530S, S1102F), *ATRX*(splice site 59571G>A, E1767K, R1372K, S79L), *BAP1*(P621L), BARD1(L341V), *BLM*(A1142V), *BRCA1*(splice site 5941G>A, P1099S), *BRCA2*(L117F), *BRD4*(P797S, P940L), *BRIP1*(Q582*), *BTK*(V415I), *C11orf30*(V356M), *CBL*(S486F), *CCND3*(Q199), *CDK12*(P1308S, S1287F), *CDK6*(P55S), *CHD2*(splice site 3595+1G>A), *CHD4*(G300D), *CREBBP*(P891S), *CTNNA1*(R27K), *CTNNB1*(splice site 1185+1G>A, A759V), *DAXX*(S580fs*74), *DICER1*(S1142F), *EP300*(D1107N), *EPHA7*(G358E), *EPHB1*(T944I), *ERBB2*(W825*), *ERBB3*(splice site 1480+1G>A), *EGFR*(P877S), *FAM123B*(D753N, E839K, P304S), *FANCA*(W22*, A1435V), *FANCC*(P346S), *FAT1*(A2637T, P4247L), *FLT1*(T91I), *FLT3*(G754E), *FRS2*(G373E), *GATA2*(S251F), *GATA3*(S199F), *GRIN2A*(D1115N), *HSP90AA1*(V453I), *IGF1R*(T345S), *IRS2*(S1327F), *JAK1*(E927K), *KDM5C*(T569I), *KDR*(A881V), *KEAP1*(G476E), *KEL*(E239Q), *LRP1B*(G3142E, G3383E, G4401D, G506D, P328S), *MAGI2*(P901S), *MDM2*(E346K), *MED12*(A240V), *MLH1*(Q26*, P648S, S44F, T45A, T82I, V664M), *MLL*(G139E, P3214S), *MLL2*(G354R, L4019F, P1048S, P4354L, S1053F, V4585I), *MLL3*(E3873K, Q1188*, V884M), *MTOR*(A2492T, P568S), *NFE2L2*(P300S), *NKX21*(P328S), *NOTCH1*(G2144D), *NOTCH2*(E459K), *NOTCH3*(A1450T, P794S0), *NPM1*(A77T), *NSD1*(A1477V), *NUP93*(E127K), *PAK3*(E319K), *PBRM1*(L203F), *PIK3CB*(R658K), *PIK3R2*(V297I), *PLCG2*(S679F), *POLE*(A341T, S891F), *PPP2R1A*(A68V, D552N), *PRDM1*(G364D), *PREX2*(R281W), *PRKDC*(D2821N), *PRSS8*(A95V,P88S,S26L), *PTCH1*(splice site 2887+1G>A, E339K, P261S), *RANBP2*(D2490N), *RARA*(G429D), *RB1*(rearrangement exon 27), *RBM10*(A618V), *RET*(G135D, T5M), *RICTOR*(R1618K, R18W), *ROS1*(P2309S), *SETD2*(L1066F, P2392L), *SMAD3*(splice site 607+1G>A),  *SMO*(P10S), *SNCAIP*(L423F), *SOX9*(P260S), *SOX10*(P269L), *SPEN*(A2765T, P2428S), *SPTA1*(D1721N), *TET2*(E1401K), *TNFAIP3*(G777S), *TSC1*(splice site 22091G>A), *TSC2*(W441*), *XPO1*(A881T), *ZNF217*(P450L) |
|  | 6 | LO | Yes, AO | 1^st^ FM sample:  FoundationOne vII  (post-treatment)  2^nd^ FM sample:  FoundationOne vII  (post-treatment) | 1^st^-2^nd^ | VIN | *BRIP1*(W448*), *CDKN2A*(bi-allelic loss), *CDKN2B*(bi-allelic loss), *CTNNA1*(G734R), *FUBP1*(R65fs*3), *IDH1*(R132H), *KDM6A*(G89E), *MAP3K1*(T949_E950insT), *MLL2*(N2965S), *MLL3*(R4594K, S1954C), *MPL*(T121I), *MTOR*(A321V, L2508I), *MUTYH*(M139I, W99*), *PDGFRA*(R718G), *SPTA1*(Q1855*), *SPEN*(G502E), *SPTA1*(Q1855*), *hTERT* promoter(-146C>T), *TET2*(G1160E, H1806R) | *ABL2*(G705E, S166N), *ACVR1B*(G344R), *ARAF*(G184S), *ARID1A*(G1942D), *ARID2*(splice site 284+1G>A), *ASXL1*(E431K, S488F), *BARD1*(L775F), *BCOR*(A105T), *BCORL1*(T1261I), *BLM*(D1071N, T80I), *BRCA2*(T203I), *CBFB*(W110*), *CDC73*(R484C), *CDH1*(R209K, S840F, V611I), *CDK12*(G1347R, L1471F), *CDKN1B*(splice site 475+1G>A), *CHD2*(P833L), *CHD4*(splice site 2121+1G>A), *CREBBP*(splice site 1330+1G>A), *CSF1R*(Q121*), *CTNNA1*(D539N), *DAXX*(P428S), *EP300*(P500L, S401L), *EPHA5*(G249E), *EPHA7*(G524E), *EPHB1*(T890I), *ERBB3*(V831M, W948*), *ESR1*(V141M), *FANCD2*(P963L), *FANCG*(R478K), *FAT1*(R1264K), *FGF10*(T7I), *FGF14*(W41*), *FGF6*(S196F), *FGFR4*(G70S), *GNAS*(P53S), *HNF1A*(Q221*), *IGF1R*(Y662C), *INPP4B*(S143N), *KDM5A*(P15L), *LRP1B*(splice site 8149+1G>A), *MAGI2*(E1253K), *MAP3K1*(S1088N), *MET*(P1073L, S103N, V437I), *MLL*(G666D, P1159L), *MLL2*(L2661F, P4920S), *MLL3*(R1945K, S1860F, T4486I), *MSH2*(D660fs*25, A53V), *MSH6*(P107L), *MYC*(S184N), *NF2*(splice site 999+1G>A), *NSD1*(E1859K), *NTRK2*(splice site 1937+1G>A), *NTRK3*(P582S), *PALB2*(A323T, L1070F, V398M), *PIK3R1*(P129S), *PIK3CA*(G505R), *PLCG2*(G705E, splice site 867+1G>A), *POLD1*(G1063D), *PRKDC*(A2882T, S2740N), *PTCH1*(S35F), *RICTOR*(P304S), *RPTOR*(E214K),  *SETD2*(A713T, P114S), *SMARCA4*(G142R), *SOX10*(P89L), *STAG2*(L237F, L345F), *SUFU*(S96N), *SYK*(S307F), *TET2*(C1271Y, E846K, S1157_M1159del), *TGFBR2*(V429I), *TNFAIP3*(P336L), *TOP1*(E255K), *U2AF1*(splice site 3491G>A), *WT1*(W486*), *ZNF703*(G162D, P379L) | *AKT3*(G407E), *ARID1B*(G466fs*30), *ATRX*(E501K), *BCOR*(G24E), *BRCA1*(G1087R), *BTK*(T339K), *CHEK2*(R132K), *DOT1L*(S132F), *EPHA3*(T215M, G955D), *EPHA7*(G796E), *ERBB4*(H1050R), *FANCD2*(E887K), *KDM5A*(G474E, G815E), *MEF2B*(G242R), *MEN1*(G536D), *MLL2*(V3787A), *MLL3*(E1240K, H2087Y), *MSH6*(T767I), *MTOR*(L1493R), *NOTCH1*(E2071K), NTRK3(T578I), PAK3(S200L), *PIK3R1*(G376R, R348*), *PDCD1LG2*(V133I), *PIK3C2B*(S156N), *PMS2*(T408I), *RAC1*(P50S), *RB1*(G893R), *SETD2*(G2343R), *SLIT2*(P926L), *SMAD2*(G122R), *SMARCA4*(G97E), *SPEN*(E2151K), *STAT3*(E552K), *TOP2A*(V1376A), *TP53*(V272M), *TSC2*(1059I), *ZNF217*(L28F) |
| Group B | 7 | GBM | NA | 1^st^ FM sample: FoundationOne vI  (pre-treatment)  2^nd^ FM sample: FoundationOne vI  (post-treatment) | 1^st^-2^nd^ | TEM, RT, BEV, PON | *APC*(I1307K), *FGFR3*(K650E) | *PIK3CA*(E542K) | *AR*(Q58_Q71del), *ARID2*(V1649L), *ATR*(G2319E), *BCL6*(Q397H), *BCOR*(E811K), *C11orf30*(S536N), *CDH1*(A137P), *CDKN2A*(Q50*), *CSF1R*(C434Y), *CTNNB1*(V358L), *DAXX*(A95T), *EP300*(S1730F), *ERBB2*(T479M), *ERBB4*(D564N), *FAT3*(V3989M), *FGF6*(S121F), *GNAS*(P356S), *IGF1R*(P558L), *KDR*(S679N), *KEAP1*(A40T), *KIT*(A87T), *MET*(A1251T, G762S), *MLL2*(P4175S, R755L), *MSH6*(W142*, P107L), *MTOR*(V972I), *MYST3*(L851F), *NCOR1*(A1228V, S1480G), *NF1*(A1596T), *NOTCH1*(N104S), *NOTCH2*(Q165L), *NSD1*(P360S, V1924M),  *PAK3*(G111R, G4S), *PDGFRB*(V722F), *PIK3C2G*(F867I), *PIK3CA*(G8D), *PTEN*(G127E), *RAD50*(A1216V), *RB1*(G449R),  *SMARCB1*(splice site 795+1G>A), *STAG2*(D198N, G66S), *TET2*(H1912R), *TP53*(P278S, A161T), *TSC1*(splice site 2975+1G>A) |
|  | 8 | GBM | NA | 1^st^ FM sample: FoundationOne vII  (pre-treatment)  2^nd^ FM sample: FoundationOne vII  (post-treatment) | 1^st^-2^nd^ | TEM, RT | *ABL2*(C25Y), *APC*(I1307K), *CDKN2A*(bi-allelic loss), *CDKN2B*(bi-allelic loss), *CREBBP*(P1053L), *EGFR*(amplification, V774M), *HSP90AA1*(S10A), *SPTA1*(R725*, P285L),  *hTERT* promoter(-124C>T) | *PIK3R1*(D540_S541ins33) | *APC*(D694_Q695>G*), *AR*(A242T), *ARID2*(G1310E, P774L), *BRAF*(N581S), *EGFR*(EGFRvII), *MLL3*(D226N), *MYST3*(P74L), *PIK3R1*(M563fs*9), *SMAD4*(G393D), *SPEN*(D1011N), *TGFBR2*(T530I) |
|  | 9 | AA | No | 1^st^ FM sample: FoundationOne vII  (pre-treatment)  2^nd^ FM sample: FoundationOneHeme  (post-treatment) | 1^st^-2^nd^ | TEM, RT | *ATM*(R1918T), *FANCD2*(K770R), *GNA13*(S22T), *JAK3*(R925S) | *EGFR*(N771_P772insN), *MDM4*(amplification), *PTEN*(D24G), *RB1*(E746fs*3), *STAG2*(R259*) | *CDKN2A*(bi-allelic loss), *CDKN2B*(bi-allelic loss), *EGFR*(amplification, G305_S306del), *KDM5A*(S1201C), *PTEN*(R173C) |
|  | 10 | LA | Yes, AA | 1^st^ FM sample: FoundationOne vII  (pre-treatment)  2^nd^ FM sample: FoundationOne vII  (post-treatment) | 1^st^-2^nd^ | TEM, RT, VOR | *ATM*(R717W), *ATRX*(S1012*), *IDH1*(R132H), *PALB2*(V78I), *PTCH1*(R945Q), *RAC1*(D90fs*20), *ROS1*(A503S), *SMARCA4*(I1055V), *TP53*(R273C) | *FGF19*(bi-allelic loss), *FGF3*(bi-allelic loss), *FGF4*(bi-allelic loss), *GNAS*(amplification), *KIT*(amplification), *NOTCH1*(amplification), *PDGFRA*(amplification, rearrangement), *QKI*(amplification), *SOX2*(amplification) | *BRAF*(D594G), *CEBPA*(G242S), *DDR2*(P42fs*32), *DOT1L*(T1068N), *EP300*(L415P), *FANCD2*(C758S), *JAK2*(R564Q), *NOTCH2*(D1306N), *NTRK2*(N415S), *PBRM1*(S995fs*1), *SETD2*(splice site 5277+2T>C) |
|  | 11 | LA | Yes, GBM | 1^st^ FM sample: FoundationOne vII  (pre-treatment)  2^nd^ FM sample: FoundationOne vII  (post-treatment) | 1^st^-2^nd^ | TEM, RT, LOM | *CTNNB1*(N287S), *EPHB1*(M18V), *FANCD2*(T61M), *JAK3*(R925S), *PTEN*(Y16*),  *hTERT* promoter(-124C>T) | NA | *CDK4*(amplification), *ERBB3*(rearrangement), *FUBP1*(D48G), *MDM2*(amplification), *SPTA1*(R785Q) |
|  | 12 | AO | No | 1^st^ FM sample: FoundationOne vII  (pre-treatment)  2^nd^ FM sample: FoundationOne vII  (post-treatment) | 1^st^-2^nd^ | TEM, RT | *ABL2*(M975I), *ATRX*(G572fs*3), *IDH1*(R132H), *NTRK1*(R750C), *SETD2*(A1124V), *SPEN*(S2306del), *TP53*(L252P) | *AR*(A766T), *ARID1A*(Q1452fs*29, Q403fs*220) | *CDKN2A*(bi-allelic loss), *CDKN2B*(bi-allelic loss), *PDGFRA*(amplification, bi-allelic deletion of exons 8 and 9, E229K) |
|  | 13 | AA | No | 1^st^ FM sample: FoundationOne vII  (pre-treatment)  2^nd^ FM sample: FoundationOne vII  (post-treatment) | 1^st^-2^nd^ | TEM, RT | *ESR1*(Y130C), *PIK3C2B*(S1041T), *PTEN*(G132A), *RANBP2*(G1587V),  *hTERT* promoter(-124C>T) | NA | *CBL*(C384Y) |
|  | 14 | GBM | NA | 1^st^ FM sample: FoundationOne vII  (pre-treatment)  2^nd^ FM sample: FoundationOne vII  (post-treatment) | 1^st^-2^nd^ | TEM, RT | *BRAF*(D22N), *CDH1*(G761R), *CDKN2A*(bi-allelic loss), *CDKN2B*(bi-allelic loss), *EGFR*(A289V, amplification), *FANCA*(R318M,S176F), *FANCD2*(N791S), *GATA6*(P54A), *GPR124*(C1196Y), *MITF*(E123K,R393Q), *MLL2*(S3708R), *NOTCH3*(G1347R), *SETD2*(S1769Y),  *hTERT* promoter(-146C>T) | *EGFR*(EGFRvIII, EGFRvIVa, E317D), *FLT3*(F942L), *IRS2*(A694_A695del), *MPL*(T183M) | *HRAS*(G13D), *KDM5C*(W93C), *IRS2*(A700_A701del) |
|  | 15 | GBM | NA | 1^st^ FM sample: FoundationOne vII  (pre-treatment)  2^nd^ FM sample: FoundationOne vII  (post-treatment) | 1^st^-2^nd^ | TEM, RT | *CDK4*(amplification), *GRM3*(A115T), *JAK3*(A373G), *PIK3R1*(Y688_S689>C), *PTCH1*(P702R), *SMO*(R173C),  *hTERT* promoter(-124C>T), *TP53*(P153fs*28) | NA | *ETVS*(rearrangement), *PTEN*(I28T) |
|  | 16 | GBM | NA | 1^st^ FM sample: FoundationOne vII  (pre-treatment)  2^nd^ FM sample: FoundationOne vII  (post-treatment) | 1^st^-2^nd^ | TEM, RT, BEV | *BRCA2*(G267E), *CDKN2A*(rearrangement, bi-allelic loss of p14ARF in exon 1), *CDKN2B*(bi-allelic loss), *EGFR*(amplification, EGFRvIII), *FAT1*(C253R, T2997M), *FLCN*(L418fs*50), *MUTYH*(Y165C), *MYCL1*(P18R), *PTEN*(bi-allelic loss), *RNF43*(R609W),  *hTERT* promoter(-146C>T) | NA | *FAS*(bi-allelic loss of exons 1 and 2, rearrangement), *FRS2*(amplification), *GPR124*(G71V), *MDM2*(amplification), *ZNF217*(rearrangement) |
|  | 17 | GBM | NA | 1^st^ FM sample: FoundationOne vII  (pre-treatment)  2^nd^ FM sample: FoundationOne vII  (post-treatment) | 1^st^-2^nd^ | TEM, RT, LAP, ACA, PEM | *ATM*(R805Q), *CDKN2A*(bi-allelic loss), *CDKN2B*(bi-allelic loss), *EGFR*(amplification), *JAK3*(R799C), *PIK3CA*(E39K), *PIK3R1*(S254C), *PIK3R2*(I135T), *hTERT* promoter(-124C>T), *TSC1*(S115F) | NA | *EGFR*(A289D, M567I, rearrangement), *ROS1*(F1871L),  *TP53*(L252P, R342P, T125A, T155N) |
|  | 18 | GBM | NA | 1^st^ FM sample: FoundationOne vII  (pre-treatment)  2^nd^ FM sample: FoundationOne vII  (post-treatment) | 1^st^-2^nd^ | TEM, RT, OPT, OSIM | *ARAF*(T181N), *CDKN2A*(bi-allelic loss of exon 1, rearrangement), *CDKN2B*(bi-allelic loss), *CTCF*(P315S), *EGFR*(amplification, EGFRvIII), *STAG2*(splice site 6682A>G), *hTERT* promoter(-124C>T) | NA | *MDM4*(amplification), *PIK3C2B*(amplification) |
|  | 19 | GBM | NA | 1^st^ FM sample: FoundationOne vII  (pre-treatment)  2^nd^ FM sample: FoundationOne vII  (post-treatment) | 1^st^-2^nd^ | TEM, RT, LAP | *CREBBP*(N1978S), *EGFR*(R222C), *FANCA*(D1033E), *KIT*(R177C), *PTEN*(Y177H, Y180C), *RANBP2*(L1076V), *TP53*(R175H) | *CDKN2A*(bi-allelic loss), *CDKN2B*(bi-allelic loss), *EGFR*(amplification),  *hTERT* promoter(-124C>T) | *TP53*(Y126*) |
|  | 20 | GBM | NA | 1^st^ FM sample: FoundationOne vII  (pre-treatment)  2^nd^ FM sample: FoundationOne Cdx  (post-treatment) | 1^st^-2^nd^ | TEM, RT | *ARID1A*(E1779G), *CDKN2A*(bi-allelic loss), *CDKN2B*(bi-allelic loss), *EGFR*(amplification), *GRM3*(Q855H), *IRF4*(F371L), *MAP3K1*(S939C), MUTYH(R520W), *hTERT* promoter(-124C>T) | *EGFR*(A289D, EGFRvIII), *JAK3*(D324fs*4), PIK3R1(G376R) | *ABL1*(R220H), *AKT1*(splice site 287+1G>A), *AKT3*(P50L), *ALK*(T1211I, T1516I), *APC*(E1295K, G2741E), *AR*(E113K), *ARAF*(T31M), *ARID1A*(P224S), *ASXL1*(S1239N), *ATM*(R1610K), *ATR*(V2520I), *AURKA*(T233I), *AXL*(P803S, V379M), *BCL2L1*(G227D), *BRCA2*(E2301K), *BRD4*(P1071L, P1127L, P908S), *BRIP1*(splice site 2257+1G>A), *C11orf30*(T912I), *CARD11*(L680F), *CBL*(E120K, G843S), *CDH1*(R321K), *CDK12*(S574N), *CDKN1B*(S125F), *CIC*(A798V, G766D), *CSF1R*(A763V), *CTCF*(E105K), *CTNNA1*(V572I), *DNMT3A*(D668N), *DOT1L*(P1231L, R1357H), *EGFR*(A289V, G719D, E391K, G810D), *EP300*(A2129T, P784S, S539F), *ERBB2*(A1165V), *ERBB3*(E555K), *ERBB4*(G1070D), *FANCC*(A226T), *FANCG*(D437N), *FBXW7*(P23L), *FGFR3*(G180S, R196H), *FGFR4*(Q41*), *FH*(G389R), *FLCN*(G322E, P298S), *FLT1*(A400V, splice site 64+1G>A), *FLT3*(A487T), *GNAS*(E201K, A188T, A269T, E268K), *GRM3*(G24E), *HGF*(P460S), *HNF1A*(S440F), *IGF1R*(G301D, V384M), *JAK3*(G892D), *JUN*(R78H, S129N), *KDM5C*(E1496K), *KDM6A*(L844F, P161S), *KEL*(A122V), *KIT*(G153E, splice site 67+1G>A), *MAP3K1*(S1131N), *MDM2*(D300N), *MED12*(G208E, P1806S), *MLL2*(splice site 5083+1G>A, G3633D, G5249S, P4155S), *MRE11A*(D619N), *MSH2*(Q451*, splice site 366+1G>A), *MTOR*(M727I, P1254L), *NF1*(A2505T, S833F), *NFKBIA*(P261L), *NOTCH2*(P224L), *NOTCH3*(A1537V), *NTRK2*(V22I), *PALB2*(H553Y), *PAX5*(A380V, P345S), *PDK1*(A391V), *PIK3C2B*(D1615N, G1109E, P103S), *POLD1*(G1001D, T131I), *POLE*(E396K), *PTEN*(T319fs*1), *QKI*(G202S, P226L), *RAF1*(L149F), *RBM10*(G172S), *RICTOR*(D1499N), *ROS1*(T299I), *SETD2*(P1439L, S566F), *SF3B1*(S1189N), *SMARCA4*(G19S, R1333Q, T950I), *SMO*(N774S, T336I), *SNCAIP*(G121D), *SOX2*(G146D), *SPOP*(G132E), *STAT3*(V671I), *STK11*(A420V), *TET2*(T1047I), *TNFAIP3*(A125T), *TP53*(A138T, R273C), *TSC2*(splice site 2743-1G>A, E157K), *ZNF217*(A564V, G792E) |
|  | 21 | GBM | NA | 1^st^ FM sample: FoundationOne vII  (pre-treatment)  2^nd^ FM sample: FoundationOne Cdx  (post-treatment) | 1^st^-2^nd^ | TEM, RT, BEV | *CDKN2A*(bi-allelic loss), *CDKN2B*(bi-allelic loss), *EGFR*(amplification, EGFRvIII), *NFE2L2*(Y366C), *PIK3CA*(P539R), *RNF43*(R609W),  *hTERT* promoter(-124C>T) | *EGFR*(D46N), GRIN2A(L267H) | *AXIN1*(V340M), *EGFR*(T263P, G239C, G810D), |
|  | 22 | GBM | NA | 1^st^ FM sample: FoundationOne vII  (pre-treatment)  2^nd^ FM sample: FoundationOne vII  (post-treatment) | 1^st^-2^nd^ | TEM, RT, OPT | *DAXX*(S647N), *FAS*(bi-allelic loss), *FAT1*(G1294R, V862I), *GNAS*(H41D), *GPR124*(C1196Y), *IGF1R*(V1195I), *KDM5A*(T269N), *LRP1B*(T3718M), *MLL2*(S4414G), *PIK3CG*(R477H), *PLCG2*(H193Q), *PTEN*(bi-allelic loss), *TP53*(C275Y) | *NF1*(R1968*, L380R), *PIK3CA*(amplification), *PIK3CG*(R784T), *SOX2*(amplification) | NA |
|  | 23 | GBM | NA | 1^st^ FM sample: FoundationOne vII  (pre-treatment)  2^nd^ FM sample: FoundationOne vII  (post-treatment) | 1^st^-2^nd^ | TEM, RT | *BRCA2*(S3366fs*4), *CDKN2A*(bi-allelic loss), *CDKN2B*(bi-allelic loss), *EGFR*(V774M), *IRS2*(G652S), *MAP3K1*(P253S), *MTOR*(M2387I),  *MYST3* (V1605G), *NF1*(R1306*),  *hTERT* promoter(-124C>T), *ZNF217*(M410V) | *PTEN*(bi-allelic loss) | *PTEN*(bi-allelic loss of exons 1 and 5) |
|  | 24 | GLIO | NA | 1^st^ FM sample: FoundationOne vII  (pre-treatment)  2^nd^ FM sample: FoundationOne vII  (post-treatment) | 1^st^-2^nd^ | TEM, PRRT | *BLM*(K323R), *CCNE1*(Y206C), *CDK4*(amplification), *ERBB3*(L1177I), *FANCA*(V750M), *FGF6*(I120V), *GLI1*(amplification), *MDM2*(amplification), *PIK3R1*(S276*), *POLD1*(A145T), *SPTA1*(F1146S), *hTERT* promoter(-146C>T) | *EGFR*(amplification), *KDR*(amplification), *KIT*(amplification), *PDGFRA*(amplification) | NA |
|  | 25 | GBM | NA | 1^st^ FM sample: FoundationOne vII  (pre-treatment)  2^nd^ FM sample: FoundationOne vII  (post-treatment) | 1^st^-2^nd^ | TEM, RT | *ALK*(M552I), *ARID1B*(A1758T), *CDKN2C*(bi-allelic loss), *EPHB1*(D577N), *MED12*(Q2086_I2087insQ), *MYC*(rearrangement in intron 1), *SPTA1*(R1757H), *TOP1*(R662Q), *TP53*(R158H) | *CDKN2A*(bi-allelic loss), *CDKN2B*(bi-allelic loss), *FANCA*(G106A, L1138V), *NF1*(bi-allelic loss exon 3 and 5), *RUNX1*(R376C), *ZNF217*(M410V) | *TSC2*(V1307fs*1) |
|  | 26 | GBM | NA | 1^st^ FM sample: FoundationOne vII  (pre-treatment)  2^nd^ FM sample: FoundationOne Cdx  (post-treatment) | 1^st^-2^nd^ | TEM, RT, PEM | *AR*(V3M), *BRAF*(D22N), *CDKN2A*(bi-allelic loss), *CDKN2B*(bi-allelic loss), *EGFR*(amplification, EGFRvIII), *FANCA*(Q1236E), GNA13(L357V), *KEL*(M1T), *MET*(L1212V), *MSH2*(V100F), *NOTCH2*(P939L) | NA | *MDM4*(amplification), *PIK3C2B*(amplification), *SPEN*(E2414fs*45) |
|  | 27 | GBM | NA | 1^st^ FM sample: FoundationOne vII  (pre-treatment)  2^nd^ FM sample: FoundationOne Cdx  (post-treatment) | 1^st^-2^nd^ | TEM, RT, PEM, OPT | *CDKN2A*(bi-allelic loss), *CDKN2B*(bi-allelic loss), *EGFR*(amplification), *FLT3*(T343I), *PALB2*(T911I),  *hTERT* promoter(-124C>T) | *EGFR*(splice site 3041_3114+185del259), *PTEN*(D326N) | *EGFR*(EGFRvIII, G312W, S220C, S227F) |
|  | 28 | GBM | NA | 1^st^ FM sample: FoundationOne Cdx  (pre-treatment)  2^nd^ FM sample: FoundationOne Cdx  (post-treatment) | 1^st^-2^nd^ | TEM, RT | *CDKN2A*(bi-allelic loss), *CDKN2B*(bi-allelic loss), *FLT1*(S364L), *MERTK*(I365V), *MTAP*(bi-allelic loss), *NF1*(R1534*), *NTRK1*(R593Q), *PTEN*(T26fs*18),  *hTERT* promoter(-124C>T), *TET2*(*1166Kext*13), TP53(C238Y) | *NF1*(truncation intron 3) | *PIK3CB*(D1067V) |
|  | 29 | GBM | NA | 1^st^ FM sample: FoundationOne Cdx  (pre-treatment)  2^nd^ FM sample: FoundationOne Cdx  (post-treatment) | 1^st^-2^nd^ | TEM, RT,  NOV, OSIM, PEM, LOM | *EGFR*(amplification, EGFRvIII), *ERCC4*(P6S), *GNA11*(rearrangement), *POLE*(R1082H), *PTCH1*(P1202H), *SGK1*(V21L), *hTERT* promoter(-124C>T) | *EGFR*(A289T, T263P), RAD54L(rearrangement) | *CDKN2A*(bi-allelic loss), *CDKN2B*(bi-allelic loss), *EGFR*(A289D), *PTEN*(M134I), *PTPN11*(K89fs*7), *SGK1*(N104S), *WT1*(amplification) |
|  | 30 | GBM | NA | 1^st^ FM sample: FoundationOne vII  (pre-treatment)  2^nd^ FM sample: FoundationACT  (post-treatment) | 1^st^-2^nd^ | TEM, RT | *PIK3CA*(E365K, H1047L) | *hTERT* promoter(-124C>T) | NA |
|  | 31 | SGA‡ | Yes, GBM | 1^st^ FM sample:  FoundationOne vI  (pre-treatment)  2^nd^ FM sample:  FoundationOne vI  (post-treatment)  3^rd^ FM sample:  FoundationOne vII  (post-treatment)  4^th^ FM sample:  FoundationOne vII  (post-treatment) | 1^st^-2^nd^ | TEM, RT | *ATM*(L2307F), *BRCA2*(C554W), *EGFR*(amplification), *MYST3*(E1107del), *TGFBR2*(S553T), *TSHR*(V721F) | *BRAF*(amplification), *CDK6*(amplification), *HGF*(amplification), *PIK3CG*(amplification), *SMO*(amplification) | NA |
|  |  |  |  |  | 2^nd^-3^rd^ | IPI, NIV | *ATM*(L2307F), *BRCA2*(C554W), *EGFR*(amplification), *MYST3*(E1107del), *TGFBR2*(S553T), *TSHR*(V721F) | NA | *FANCF*(L242fs*18), *STAT4*(R705*) |
|  |  |  |  |  | 3^rd^-4^th^ | NER, LOM, ERL | *ATM*(L2307F), *BRCA2*(C554W), *EGFR*(amplification), *FANCF*(L242fs*18), *GRM3*(amplification), *KEL*(M1T), *MYST3*(E1107del), *STAT4*(R705*), *TGFBR2*(S553T), *TSHR*(V721F) | *EGFR*(rearrangement) | *CDKN2A*(bi-allelic loss), *CDKN2B*(bi-allelic loss), *EGFR*(deletion exon 16), *FGFR4*(K387R), *MLL2*(I2073V,S606P), *MTOR*(S2215F), *NOTCH2*(K849fs*6), *PIK3CA*(H1047R), *SMO*(amplification),  *hTERT* promoter(-124C>T) |
|  | | | | | | | **Mutational similarities**  ***Gene*(mutation)** | **Unique mutations in 1^st^ FM sample**  ***Gene*(mutation)** | **Unique mutations in 2^nd^ FM sample**  ***Gene*(mutation)** |
| Group C | 32 | AA | Yes, GBM | 1^st^ FM sample:  FoundationOne vII^†^  (post-treatment)  2^nd^ FM sample:  FoundationOne vII^†^  (post-treatment) | 1^st^-2^nd^ | NA | *ARID1A*(Q561*), *ATM*(S1691R), *ATRX*(K1045fs*1), *C17orf39*(P92L), *CARD11*(A687V), *GPR124*(G71W), *HGF*(S331T), *IDH1*(R132H), *KDM5A*(amplification), *PDCD1LG2*(E11fs*7), *PRDM1*(A408P), *TP53*(R248Q, R342*) | *CCND2*(amplification), *CHD4*(V638I), *FGF23*(amplification), *FGF6*(amplification), *PTEN*(P89fs*3) | NA |
|  | 33 | AA | No | 1^st^ FM sample: FoundationOne vII^††^  (pre-treatment)  2^nd^ FM sample: FoundationOne vII^††^  (pre-treatment) | 1^st^-2^nd^ | NA | *BCL2*(I19L), *CREBBP*(Y1204F), *DNMT3A*(G543fs*108), *FANCA*(E961A), *FAT1*(D3317N), *LRP1B*(P1387S), *MAP3K1*(Q525*), *MLL3*(Q2177R), *PIK3CA*(E545K), *PTCH1*(V355fs*12), *RANBP2*(T1478P),  *hTERT* promoter(-146C>T) | *ASXL1*(G646fs*12), *CBL*(H42_L43insH), *PTCH1*(amplification), *SOX2*(amplification), *ZNF703*(A401_H402insPTHLGGSSCSTCSA) | *CBL*(L43_S44insHLS), *ZNF703*(H402_D403>PTHLGGSSCSTCSAHD) |
|  | 34 | LA | Yes, AA | 1^st^ FM sample: FoundationOne vII  (pre-treatment)  2^nd^ FM sample: FoundationOne vII  (pre-treatment) | 1^st^-2^nd^ | NA | *IDH1*(R132H), *KDR*(A824G), *MAP3K1*(S939C), *MLL3*(R841W), *MUTYH*(G382D), *NF2*(M205T), *NTRK2*(T34A), *POLD1*(F34L), *TP53*(R342*, V157F) | *EGFR*(T263P) | *ATRX*(R1426*) |
|  | 35 | GBM | NA | 1^st^ FM sample:  FoundationOne vII  (pre-treatment)  2^nd^ FM sample:  FoundationOne vII  (pre-treatment) | 1^st^-2^nd^ | NA | *ARID1A*(A344V), *BARD1*(P358_S364del), *BCOR*(E1076K), *ERBB2*(E930D), *FANCA*(T126R), *INHBA*(R229Q), *MAP3K1*(D1170Y, S939C), *PRKDC*(L3584F), *TGFBR2*(S553T), *TNFRSF14*(T253I) | *EGFR*(amplification), *PTEN*(S59*) | *CDKN2A*(bi-allelic loss), *CDKN2B*(bi-allelic loss), *CDKN2C*(bi-allelic loss), *KRAS*(G12A), *PTEN*(C124fs*2), *PTPN11*(A72D),  *hTERT* promoter(-124C>T), |
|  | 36 | OA | Yes, AA | 1^st^ FM sample:  FoundationOne vII  (pre-treatment)  2^nd^ FM sample:  FoundationOne Cdx  (pre-treatment) | 1^st^-2^nd^ | NA | *ATRX*(splice site 4121-2A>G), *BCORL1*(L84I), *CDKN2B*(D86N), *HRAS*(R153C), *IDH1*(R132H), *MSH3*(Y465C), *TP53*(R248W, Y234_N235insTTIHY) | *ATRX*(P909fs*61), *ARID1B*(G319del), *TP53*(R273C) | NA |

Definition of Groups: Group A-Patients who only had post-treatment samples analyzed by Foundation Medicine (Patient 1, 3, and 4 received radiation and temozolomide and patient 2 only received radiation) and received intervening treatments between their sample analyses, Group B-Patients who had one pre-treatment sample and received intervening treatments between their sample analyses, Group C-Patients who did not have intervening treatments between their sample analyses (Tumor samples from Patient 32 and 33 came from the same surgery)

Diagnoses: MENG-Meningioma, GBM-Glioblastoma, Grade IV; GLIO-Gliosarcoma, Grade IV; AA-Anaplastic Astrocytoma, Grade III; AO-Anaplastic oligodendroglioma, Grade III; AOA-Anaplastic Oligoastrocytoma, Grade III; LA-Astrocytoma, Grade II; LO-Oligodendroglioma,

Grade II; OA-Oligoastrocytoma, Grade II; PXA-Pleomorphic xanthoastrocytoma, Grade II; SGA-Subependymal giant cell astrocytoma, Grade I

Intervening treatments: TEM-temozolomide, RT-radiation, VIN-vincristine, NOV-Novocure TTF, OSIM-Osimertinib, PEM-Pembrolizumab, LOM-Lomustine, OPT-Optune TTF, PRRT-Peptide receptor radionuclide therapy, ACA-Acalabrutinib, LAP-Lapatinib,

VOR-Vorasidenib, CARB-Carboplatin, BEV-Bevacizumab, PON-Ponatinib, IPI-Ipilimumab, NIV-Nivolumab, NER-Neratinib, ERL-Erlotinib, TRAM-Trametinib

†Samples came from the same surgery, but different tumor blocks

††Samples came from the same surgery, same tumor block

NA-Not applicable
