# Supplementary material for: Targeted next-generation sequencing of 565 neuro-oncology patients at UCLA: A single-institution experience
Source: Neurooncol Adv. 2020 Jan 29;2(1):vdaa009. doi: 10.1093/noajnl/vdaa009 (PMC7034640; doi:10.1093/noajnl/vdaa009)
Supplement: vdaa009_suppl_Supplemental_Table_S4 [file vdaa009_suppl_supplemental_table_s4.docx]

| **Supplemental Table S4. Summary of differences and retention in mutations for glioma patients who received intervening**  **treatments between multiple Foundation Medicine tests** | | | | |
| --- | --- | --- | --- | --- |
|  | Commonly affected genes | Retained mutations:  [specific mutation(s)]  (frequency of incidence in patients) | Lost mutations:  [specific mutation(s)]  (frequency of incidence in patients) | Gained mutations:  [specific mutation(s)]  (frequency of incidence in patients) |
| Group A (n = 4 patients) | *ARID1A* | NA | [G1942D](1) | [Q1364*, Q2176fs*48]^†^(1),  [A1413V, splice site 21621G>A]^†^(1) |
|  | *ARID1B* | [Q129_Q130insQ](1), [C878R](1) | NA | [G316E] (1), [G466fs*30](1) |
|  | ATRX | [R808Q](1), [A238fs*19](1), [V2189A](1) | NA | [splice site 59571G>A, E1767K, R1372K, S79L]^†^(1), [E501K](1) |
|  | *IDH1* | [R132H](4) | NA | NA |
|  | *TP53* | [S127Y](1), [Q167fs*3](1) | NA | [V272M](1) |
| Group B (n = 25 patients) | *CDKN2A* | [bi-allelic loss](9),  [rearrangement, bi-allelic loss of exon 1]^†^(1), [rearrangement, bi-allelic loss of p14ARF in exon 1]^†^(1) | [bi-allelic loss](2) | [bi-allelic loss](4), [Q50*](1) |
|  | *CDKN2B* | [bi-allelic loss](11) | [bi-allelic loss](2) | [bi-allelic loss](4) |
|  | *EGFR* | [amplification, V774M]^†^(1),  [amplification, A289V]^†^(1),  [amplification, EGFRvIII]^†^(5),  [amplification](4), [R222C](1), [V774M](1) | [N771_P772insN](1),  [EGFRvIII, EGFRvIVa, E317D]^†^(1),  [amplification](2), [A289D, EGFRvIII](1), [D46N](1),  [splice site 3041_3114+185del259](1), [A289T, T263P](1), [rearrangement](1) | [EGFRvII](1), [amplification, G305_S306del](1),  [A289D, M567I, rearrangement]^†^(1), [A289V, G719D, E391K, G810D]^†^(1), [T263P, G239C, G810D]^†^(1),  [EGFRvIII, G312W, S220C, S227F](1),  [A289D](1), [deletion exon 16](1) |
|  | *PTEN* | [Y16*](1), [G132A](1), [bi-allelic loss](2), [Y177H, Y180C]^†^(1), [T319fs*1](1), [T26fs*18](1) | [D24G](1), [bi-allelic loss](1), [D326N](1), | [G127E](1), [R173C](1), [I28T](1),  [bi-allelic loss of exon 1 and 5](1), [M134I](1) |
|  | *hTERT* promoter | [-124C>T](12), [-146C>T](3) | [-124C>T](2) | [-124C>T](1) |

†Mutations of this gene were all present in one patient
