# Supplementary material for: Targeted next-generation sequencing of 565 neuro-oncology patients at UCLA: A single-institution experience
Source: Neurooncol Adv. 2020 Jan 29;2(1):vdaa009. doi: 10.1093/noajnl/vdaa009 (PMC7034640; doi:10.1093/noajnl/vdaa009)
Supplement: vdaa009_suppl_Supplemental_Table_S5 [file vdaa009_suppl_supplemental_table_s5.docx]

| **Supplementary Table S5: Summary characteristics of the primary *IDH* wild type glioblastoma patient cohort (n=228)** | |
| --- | --- |
| Age at glioblastoma diagnosis, mean +/- s.d. (years) | 56.5 +/- 12.5 |
| Gender, n (%) | |
| Male | 137 (60.1%) |
| Female | 91 (39.9%) |
| Karnofsky performance status, mean +/- s.d. | 85 +/- 9 |
| *MGMT* status, n (%) | |
| Methylated | 72 (31.6%) |
| Unmethylated | 156 (68.4%) |
| Extent of tumor resection, n (%) | |
| 0-10% | 23 (11.1%) |
| 10-90% | 98 (46.7%) |
| <90% | 107 (41.8%) |
| s.d.: standard deviation | |
